# Targeting Lipid Rafts—A Potential Therapy for COVID-19

**DOI:** 10.3389/fimmu.2020.574508

**Published:** 2020-09-29

**Authors:** Dmitri Sviridov, Yury I. Miller, Rami A. Ballout, Alan T. Remaley, Michael Bukrinsky

**Affiliations:** ^1^Baker Heart and Diabetes Institute, Melbourne, VIC, Australia; ^2^Department of Biochemistry and Molecular Biology, Monash University, Clayton, VIC, Australia; ^3^Department of Medicine, University of California, San Diego, La Jolla, CA, United States; ^4^Lipoprotein Metabolism Section, Translational Vascular Medicine Branch, National Heart, Lung and Blood Institute (NHLBI), National Institutes of Health, Bethesda, MD, United States; ^5^Department of Microbiology, Immunology and Tropical Medicine, School of Medicine and Health Sciences, The George Washington University, Washington, DC, United States

**Keywords:** COVID-19, SARS-CoV-2, coronavirus, lipid rafts, inflammation, lipids, cholesterol

## Abstract

COVID-19 is a global pandemic currently in an acute phase of rapid expansion. While public health measures remain the most effective protection strategy at this stage, when the peak passes, it will leave in its wake important health problems. Historically, very few viruses have ever been eradicated. Instead, the virus may persist in communities causing recurrent local outbreaks of the acute infection as well as several chronic diseases that may arise from the presence of a “suppressed” virus or as a consequence of the initial exposure. An ideal solution would be an anti-viral medication that (i) targets multiple stages of the viral lifecycle, (ii) is insensitive to frequent changes of viral phenotype due to mutagenesis, (iii) has broad spectrum, (iv) is safe and (v) also targets co-morbidities of the infection. In this Perspective we discuss a therapeutic approach that owns these attributes, namely “lipid raft therapy.” Lipid raft therapy is an approach aimed at reducing the abundance and structural modifications of host lipid rafts or at targeted delivery of therapeutics to the rafts. Lipid rafts are the sites of the initial binding, activation, internalization and cell-to-cell transmission of SARS-CoV-2. They also are key regulators of immune and inflammatory responses, dysregulation of which is characteristic to COVID-19 infection. Lipid raft therapy was successful in targeting many viral infections and inflammatory disorders, and can potentially be highly effective for treatment of COVID-19.

## Introduction

COVID-19 is the biggest global pandemic of the 21st century and it may not be the last. Increased interactions of humans with animals amplifies chances of animal viruses “jumping” to humans, while increased density of human population and abundant international travel further contribute to the extremely fast spread of the infectious diseases around the globe. At the peak of a pandemic, public health measures provide the most effective protection against spread of the infection, but when the peak passes, it leaves behind important problems. First, the virus continues circulating in the population causing clusters of outbreaks and “second waves.” Second, initial infection often causes an outbreak of various chronic diseases ensuing from the presence of a “suppressed” virus or as a long-term consequence of the initial exposure. Global immunization provides a radical solution, but full eradication of an infectious disease has been achieved only a few times throughout history. Many viruses are resistant to vaccination through rapid mutagenesis, like influenza viruses (1), employing cell-to-cell transmission altogether bypassing exposure to antibodies, like HTLV (2), gaining entry through the respiratory tract delaying the access of antibodies to the site of infection, like coronaviruses (3), or a combination of these and yet unknown factors, such as with HIV and influenza. Development of an effective vaccine against coronavirus is challenging; while animal vaccines exist, no human vaccine against any of coronaviruses has been developed so far (4). Furthermore, due to animal origin of SARS-CoV-2, existing and new animal reservoirs will provide plentiful opportunities for the virus to mutate and reemerge. Collectively, it makes it unlikely that this virus will be eradicated through vaccination, at least in the short and medium terms. A solution is to develop an anti-viral medication. Ideally, such treatment should target an early stage of the viral lifecycle, be insensitive to frequent changes of viral phenotype due to mutations (e.g., targeting host cell rather than the virus) and be safe. Preferably, this treatment should also target complications of the infection. In this Perspective, we discuss an approach that fulfills these requirements, “lipid raft therapy,” that potentially can be applied for treatment of COVID-19.

## Lipid Rafts and Viral Infections

Lipid rafts are solid domains of plasma membrane embedded into predominantly fluid membrane (5). Proteins that work together (e.g., in multiunit receptors or endocytosis machinery) are usually located in lipid rafts preventing these molecules from drifting apart, instead keeping them in proximity to each other. Lipid rafts host many receptors involved in immune and inflammatory responses and play a key role in regulation of inflammation (6), an important attribute given the role of unique pattern of immune and inflammatory responses in the clinical manifestations of COVID-19 (7, 8). At the same time, numerous viruses, e.g., HIV and Influenza virus, use host lipid rafts as a “point of entry,” owing to rafts harboring high concentration of receptors utilized to bind and guide pathogen, as well as affiliated endocytosis machinery ready to take an obligate intracellular parasite inside. Lipid rafts also serve as a platform for pathogen's assembly (e.g., HIV) and as a “point of exit” [Ebola virus, HIV and HBV (9)]. Furthermore, viruses often exploit host raft-associated pathways and modify lipid rafts through binding to rafts and/or releasing raft-modifying factors to further promote their infection cycle. The list of viruses where disruption of rafts was shown to inhibit virus infectivity is long and includes HIV, HCV, Influenza A, Ebola, Marburg and many other viruses [for review see (9, 10)]; SARS-CoV-2 may also be one of such viruses. It is however important to recognize that despite a success of this experimental approach, so far, no drug acting principally through disruption of lipid rafts has been approved for clinical use as an anti-viral treatment.

## Lipid Rafts and Pathogenesis of SARS-CoV-2

**Figure 1:**
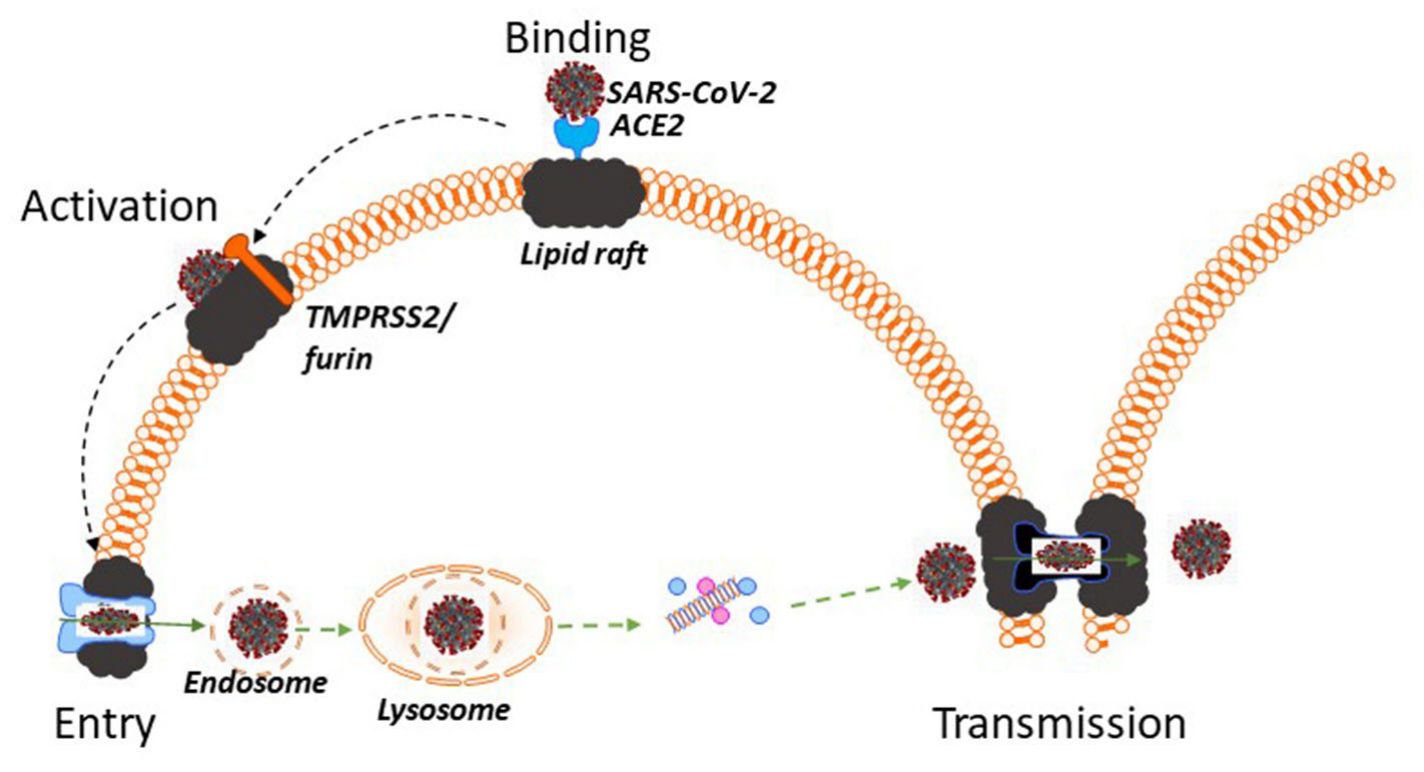
Lipid rafts and pathogenesis of SARS-CoV-2. SARS-CoV-2 docks onto ACE2, which is a lipid raft protein. After binding to ACE2 the S protein in the viral envelope undergoes enzymatic activation by TMPRSS2 or furin, which are likely located in lipid rafts. Subsequent endocytosis of SARS-CoV-2 occurs using raft-dependent endocytic pathway. After internalization SARS-CoV-2 undergoes intracellular trafficking within endosomes, fuses with mature lysosomes and releases its viral RNA genome into the host cytoplasm. One of the pathways of virus transmission, cell-to-cell transmission, occurs through formation of intercellular channels or syncytia and also requires intact lipid rafts. Thus, at least four stages of SARS-CoV-2 lifecycle, initial binding, activation, internalization and cell-to-cell transmission, require intact host rafts to proceed and, if other viruses are a guide, disruption of lipid rafts using lipid raft therapy mitigates the infection.

Molecular pathogenesis of SARS-CoV-2 is schematically presented in Figure 1. SARS-CoV-2 is very similar to its close relative SARS-CoV and pathogenic pathways of both viruses interact with pathways of cellular cholesterol metabolism (11). Both viruses carry a spike (S) protein in their envelope, which is essential for entry into the host cells (12). The S protein docks the viral particle onto angiotensin-converting enzyme 2 (ACE2) (12, 13), a membrane protein particularly abundant in the plasma membrane of type II pneumocytes, nasal goblet secretory cells and enterocytes (12, 14). ACE2 is a lipid raft protein; disruption of lipid rafts prevents its correct exposure making it impossible for the virus to dock (15, 16). After binding to ACE2, the S protein must undergo enzymatic conversion (activation) by either the transmembrane serine protease 2, TMPRSS2, or furin (12). The exact location of these proteases on the plasma membrane is unknown, however, TMPRSS2 co-localizes with ACE2 and is potentially palmitoylated (17), indicating likely lipid raft localization. Cleavage-induced conformational change in the S protein and ACE2 allows the host cell membrane to invaginate, which is essential for initiating endocytic viral entry. Endocytic and fusion pathways used by SARS-CoV to enter the cell rely on a lipid raft-specific machinery and lipid-raft localization is essential for them to function (18). After internalization, the virus undergoes intracellular trafficking within endosomes, which eventually fuse with mature lysosomes. Within the lysosome, the S protein undergoes another series of enzymatic cleavages and modifications, followed by release of the viral RNA genome into the host cytoplasm (18). Furthermore, an important feature of both SARS-CoV (19) and SARS-CoV-2 (20) is an ability for cell-to-cell transmission, which allows the virus to escape contact with antibody. Cell-to-cell transmission through formation of channels or syncytia requires intact lipid rafts (21). Thus, at least four stages of SARS-CoV-2 lifecycle, initial binding, activation, internalization and cell-to-cell transmission, require intact host rafts to proceed (Figure 1). It follows that targeting host lipid rafts may be an effective strategy to reduce infectivity of SARS-CoV-2, and this was experimentally shown *in vitro* for SARS-CoV (22).

## Raft Therapeutics

Targeting lipid rafts for treatment of various diseases, from neurodegeneration and neuropathic pain to cancer, infections and atherosclerosis, is a rapidly growing therapeutic approach, as described in detail in our recent review (23). Fundamentally, there are two ways to utilize lipid rafts for anti-viral therapy, either to directly disrupt these domains, or to use lipid raft for targeted delivery of anti-viral therapeutics. As only approaches involving lipid raft disruption have been investigated in the context of SARS-CoV-2 pathogenesis (Figure 1), we will primarily focus on this mechanism.

One approach is to reduce abundance of lipid rafts by depleting them of lipids responsible for their stability, cholesterol or sphingomyelin. For example, cholesterol can be physically removed from rafts by sequestrants, such as cyclodextrins. β-Cyclodextrin is used as a food additive and is proven to be safe for consumption (24), it inhibits entry of another enveloped virus, HIV, into host cells (25) and has anti-inflammatory properties (26). Another option is stimulation of physiological pathways responsible for the efflux of cholesterol, such as stimulation of the expression of ATP-binding cassette transporters A1 and G1, using LXR agonists or heterologous over-expression of these transporters. Further, elevation of the levels of natural acceptors of cholesterol, high-density lipoprotein (HDL) and apolipoprotein A-I (apoA-I), or infusion of HDL and apoA-I mimetics also reduces the abundance of lipid rafts. The third option is to reduce cholesterol and sphingolipid supply using inhibitors of their biosynthesis, e.g., statins and Miglustat, respectively. Given the proven safety record of statins, drugs widely used to treat hypercholesterolaemia, they are a good candidate for immediate testing for this application. Finally, recently discovered modulator of lipid rafts, apoA-I Binding Protein (AIBP), may be an especially beneficial “lipid raft therapy” compound to treat COVID-19. AIBP stimulates cholesterol efflux and reduces the abundance of lipid rafts in various tissues (27–29), it effectively reduces inflammation (29) and was recently shown to have an anti-HIV activity (30). Importantly, AIBP targets only cells activated with LPS, cholesterol-loaded or infected, reducing the abundance of lipid rafts to the “healthy level,” but not below (28, 30), a selectivity beneficial for avoiding adverse side-effects. Another advantage of AIBP is that it remains active when administered directly to lungs via inhalation (31), an application especially relevant to COVID-19.

Second approach takes advantage of unique partitioning of various receptor assemblies to the lipid rafts allowing for an effective targeting of therapeutic agents specifically to these domains. Lipid-coated nanoparticles (32) or liposomes carrying a vector (e.g., antibody to ACE2 or another raft protein) can carry anti-viral therapies, such as Irbesartan, an ACE2 antagonist, Camostat mesylate, a TMPRSS2 inhibitor, or heparin, which breaks down proteoglycans essential for the SARS-CoV-2 binding to the cell, thus increasing the treatment efficiency and reducing the effective drug concentration and toxicity. Finally, β-cyclodextrin (33) and synthetic HDL (34, 35) particles can also be used to deliver to cells compounds with limited solubility, such as Remdesivir.

## Lipid Rafts and Co-Morbidities of COVID-19

One of the puzzling features of COVID-19 infection is abnormal immune response (7) and severe inflammation resembling autoimmune vasculitis (36, 37) or sepsis (38). The exact pathogenesis of dysregulation of the immune and inflammatory responses in COVID-19 is unknown and emergency response relies on general anti-inflammatory medications or inhibitors of inflammatory cytokines. These approaches in most cases are effective in mitigating the acute phase, but, if sepsis and autoimmune vasculitis are a guide, heightened chronic inflammation will persist for a long time causing multiple chronic co-morbidities. Lipid rafts play a key role in immunity and inflammation (6) and targeting lipid rafts to reduce inflammation is a promising therapeutic approach that can reduce both acute and chronic inflammatory responses (23). Another common co-morbidity of COVID-19 is coagulopathy (39). Lipid rafts are involved in regulation of platelet function and lipid raft therapy was effective in attenuating platelet function (40). Thus, therapeutic approaches targeting lipid rafts may mitigate both COVID-19 infection itself and its acute and chronic co-morbidities.

## Discussion

Use of “lipid raft therapy” approach has important advantages over other strategies. First, it targets the element of pathogenesis common to both acute and residual infection, as well as the most likely co-morbidities of COVID-19. Second, lipid rafts are a cellular element resistant to rapid mutagenesis, their involvement in pathogenesis of viral infection is common to many viruses and the proposed treatments may be applicable for both current and future pandemics. Third, it utilizes medications that are currently used or being tested for treatment of other diseases, where therapeutic doses were proven to be safe and have high degree of hemocompatibility promising a rapid repurposing for treatment of COVID-19. Finally, it can be used both as a stand-alone therapy and in combination with other therapeutic approaches.

Lipid raft therapy also has some limitations. Agents of lipid raft therapy may trigger immune response, which may dangerously combine with the abnormal immune response to the SARS-CoV-2. Although no adverse immune reaction to any of the lipid raft therapy agents has been described so far (even to large multiprotein complexes such as HDL), such reaction in the context of COVID-19 infection cannot be *a priori* dismissed. Conversely, disruption of rafts reduces immune responses, which may have a negative effect on resistance to the virus. However, several lines of evidence suggest that suppressed immunity is not detrimental, but could be even beneficial for the anti-COVID-19 therapy. Immunosuppression and chemotherapy did not have a negative effect on severity of COVID-19 (41, 42). Asymptomatic patients have lower levels of virus-specific antibody than those with severe disease manifestation (43). In humans, ACE2, the receptor for SARS-CoV-2, is stimulated by interferon, a key element of anti-viral immune response (14). Collectively, these findings suggest that SARS-CoV-2 is hijacking immune responses, and mitigation of immune response, perhaps counterintuitively, may be beneficial. Another limitation is that, given that lipid rafts are a central element of numerous signaling pathways, their disruption may have unintended negative consequences for functionality of these pathways. However, this complication is more of a theoretical nature: while phenotype of excessive raft abundance has well-described manifestations, very little is known of a phenotype of lipid raft deficiency (23), most likely due to a high level of redundancy in regulation of lipid raft originating pathways. Furthermore, some therapeutic approaches, such as AIBP, allows “fine tuning” of the rafts, reducing the overabundant lipid rafts to the “normal” level, but not below (28, 30). Finally, some agents of lipid raft therapy may have side-effects seemingly unrelated to their activity toward lipid rafts. For example, high doses of hydroxypropyl-β-cyclodextrin had unexpected impact on renal and systemic hemodynamics (44). These potential toxicities should be carefully considered when designing therapeutic regimens.

## Author's Note

After this paper has been accepted for publication, the following article has been published also emphasizing the role of lipid rafts in the pathogenesis of COVID-19 (45).

## Author Contributions

DS conceptualized the manuscript and wrote the first draft. YM, RB, AR, and MB contributed ideas and edited subsequent versions on the text. All authors contributed to the article and approved the submitted version.

## Conflict of Interest

DS, YM, and MB are inventors listed in patent applications related to the topic of this paper. YM is scientific co-founder of Raft Pharmaceuticals LLC. The terms of this arrangement have been reviewed and approved by the University of California, San Diego in accordance with its conflict of interest policies. The remaining authors declare that the research was conducted in the absence of any commercial or financial relationships that could be construed as a potential conflict of interest.
